# The effect of aging on the translucency of contemporary zirconia generations: in-vitro study

**DOI:** 10.1186/s12903-024-04465-6

**Published:** 2024-06-27

**Authors:** Aya A. Salama, Karim A. Shehab, Sherif Samir Bushra, Faisal Safwat Hamza

**Affiliations:** 1 Department of Fixed Prosthodontics, October University for Modern Sciences and Arts, Giza, Egypt; 2 Lecturer of Orthodontics, October University for Modern Sciences and Arts, Giza, Egypt

**Keywords:** Monolithic Zirconia, Thermocycling, Accelerated aging, Esthetics

## Abstract

**Background:**

The translucency of different zirconia generations at each time point after thermocycling aging is still lacking.

**Methods:**

Four zirconia materials were used with a total of 60 samples produced from monolithic third generation (5Y) 5 mol% yttria-stabilized zirconia polycrystalline ceramic and fourth generation zirconia (4Y) 4 mol% yttria-stabilized zirconia polycrystalline ceramic, represented by [group1:[CM-5Y] Ceramill Zolid fx (3rd generation zirconia) (Amann Girrbach, Koblach, Austria), group 2:[CM-4Y] Ceramill Zolid HT + (4th generation zirconia) (Amann Girrbach, Koblach, Austria), group 3:[CC-5Y] Cercon XT/ML (Dentsply Sirona, Germany) (3rd generation), and group 4:[CC-4Y] Cercon HT/ML (Dentsply Sirona, Germany) (4th generation)]. The L*a*b* figures were measured by using a spectrophotometer at baseline and after 10,000, 30,000, and 50,000 cycles of thermocycling. At each interval, the translucency of the samples was estimated by using the translucency formula CIEDE2000. The Scheffe post-hoc compared differences among each of the four materials. The Repeated measures ANOVA tested the differences between the materials at each of the different thermocycling intervals (*p* < .001). Data analyses were evaluated at a significance level of *p* < .05 (CI 95%).

**Results:**

Two-way ANOVA revealed that at baseline the third and fourth generation’s zirconia showed statistically significant differences in translucency (*P* < .001). Translucency values at baseline and after thermocycling exhibited statistically significant changes (*p* = .003). At each of the time interval; CM-4Y had the highest translucency values followed by CM-5Y, CC-4Y and CC-5Y had the least translucency values.

**Conclusions:**

The third and fourth generations of zirconia displayed different translucencies. Thermocycling affected the translucency of both third and fourth generations of zirconia. At each of the time intervals group 2:[CM-4Y] had the highest TP followed by group1:[CM-5Y], while, group 3:[CC-5Y] and group 4:[CC-4Y] had the least TP.

## Introduction

Two of the most crucial factors that practitioners evaluate when deciding on dental ceramic materials are esthetics and durability [1]. The Translucency of ceramics is considered a parameter of esthetics and is essential for mimicking the appearance of teeth. Zirconia is currently one of the most popular ceramics on the market. Despite its greatly enhanced flexural strength, the absence of a glass phase in zirconia diminishes its translucency when utilized as monolithic zirconia restorations, which is an aesthetic concern [1, 2].

Manufacturers currently claim that zirconia’s translucency has improved thanks to modern material compositions which lead to improved aesthetics while keeping the majority of its strongest qualities. By increasing the yttrium proportion, it is stated that the translucency of contemporary zirconia materials has been enhanced [2, 3]. The translucency of monolithic zirconia restorations has also been enhanced by other means. Manufacturers have taken an approach by creating zirconia blanks with multiple, differently colored layers [3]. According to their mechanical and optical properties, zirconia materials are classified into four generations [2, 4].

Initially, first-generation zirconia with 3 mol% yttria-stabilized tetragonal zirconia polycrystals (3Y-TZP), also known as conventional zirconia was recommended as a core material for fixed dental prostheses in conjunction with a more aesthetically pleasing feldspathic porcelain veneer. Nonetheless, this multilayer zirconia prosthesis was reported to have substantial chipping rates [4, 5]. This type of zirconia has a high refractive index. The material is optically opaque due to the extremely high number of interfaces [3].

To enhance the aesthetics of zirconia polycrystalline ceramic, the alumina additive concentration was decreased, and the ceramic was sintered at higher temperatures [5, 6]. These changes gave rise to the 2nd generation of 3Y-TZP ceramic with reduced alumina content and enabled the production of monolithic posterior prostheses. The third generation of zirconia-based ceramics was created in order to achieve greater esthetics. This generation’s cubic phase concentration was raised by adding extra stabilizing oxides, resulting in stabilized zirconia containing 5 mol% yttria. Although the material’s translucency was enhanced, its strength and fracture toughness were degraded because cubic grains cannot undergo phase transformation under stress [6, 7].

4th generation multi-chromatic zirconia with 4 mol% yttrium with shade and translucency gradients was intended to enhance both the mechanical and optical qualities of monolithic zirconia. Third and fourth zirconia generations are produced as partially stabilized (PSZ) or fully stabilized (FSZ) materials [7, 8]. With the most recent generation starting to be produced by some manufacturers, the fifth generation is now on market, the highly translucent zirconium oxide of the next generation now offers natural shade gradient matching and uniformly high strength. The newest generation’s member is made from yttria zirconia and continues to go through rigorous quality assurance testing in the manufacturing companies [7, 8]. Dental laboratories are currently using third and fourth generation zirconia the most in the production of fixed dental prostheses [2]. However, it still had the limitation of a lower translucency parameter (TP) than glass ceramics [9–12].

The translucency of a material is affected by its color, thickness, background color and surface texture [13, 14]. However, it is highly subjective to fail intraorally due to the fatiguing of ceramic material over time in the humid oral environment. Testing ceramic materials in conditions simulating the oral environment as thermocycling is required to mimic fatigue processes experienced by materials in the oral environment, which might compromise the aesthetic outcome [15–18].

The null hypothesis of this study was that there would be no difference in the translucency among the different types of zirconia tested within each time interval after thermocycling.

## Methods

This in-vitro study was approved by the Institutional Ethics Committee of the University (No.ETH: 223). By adopting an alpha (α) level of 0.05 (5%), a beta (β) level of 0.05 (5%), i.e., power = 95%, and by using the G-Power Sample Power Calculator, a power analysis was determined (Universitat Kiel, Kiel, Germany). With an effect size of 0.8, a total sample size of 60 (*n* = 15 each group) was necessary based on the findings of Jerman et al. [15].

For this study, four different zirconia materials were chosen, with 15 samples for each type. The chosen materials were group1: [CM-5Y] Ceramill Zolid fx (3rd generation zirconia) (Amann Girrbach, Koblach, Austria), group 2: [CM-4Y] Ceramill Zolid HT + (4th generation zirconia) (Amann Girrbach, Koblach, Austria), group 3: [CC-5Y] Cercon XT/ML (Dentsply Sirona, Germany) (3rd generation), and group 4: [CC-4Y] Cercon HT/ML (Dentsply Sirona, Germany) (4th generation) (Table 1). For both Ceramill and Cercon discs, the color shade was A1.

**Table 1:** Third and fourth Zirconia generations information

Brand Name	Batch (Lot) number	Chemical composition	Sintering time, and temperature	Manufacturer
Ceramill Zolid fx	1,904,000	•Zirconium oxide •Yttrium oxide 8.5–9.5%. •Hafnium oxide HfO_2_ < 5% •Aluminum oxide Al_2_O_2_, Silicon oxide, other oxides < 1%	Ceramill Therm, 2 h, 1450 °C	Amann Girrbach, Koblach, Austria
Ceramill Zolid HT +	2,004,001	• Zirconium oxide • Yttrium oxide 6.7–7.2% • HfO_2_ < 5% • Al_2_O_2_, Silicon oxide, other oxides < 1%	Ceramill Therm, 5 h, 1450 °C	Amann Girrbach, Koblach, Austria
Cercon XTML	18,041,180	• Zirconium oxide • Yttrium oxide 9% • HfO_2_ < 3% • Al_2_O_2_, Silicon oxide, other oxides < 2%	inLab Profire, Speed sintering in 2 h and 50 min, 1500 °C	Dentsply Sirona, Germany
Cercon HTML	18,041,192	•Zirconium oxide • Yttrium oxide 5 – 9% • HfO_2_ < 3% • Al_2_O_2_, Silicon oxide, other oxides < 2%.	inLab Profire, Speed sintering in 2 h and 50 min, 1500 °C	Dentsply Sirona, Germany

Blocks were milled from the original circular discs, following the dimensions of 14 mm (length L), 16 mm (width W), 1 mm (height H). The zirconia blocks were then sectioned using a water-cooled diamond disc (Isomet 4000 linear precision saw, Buehler Ltd., Lake Bluff, IL). The 20% reduction in size expected after sintering was factored into the cutting dimensions.

**Fig. 1:**
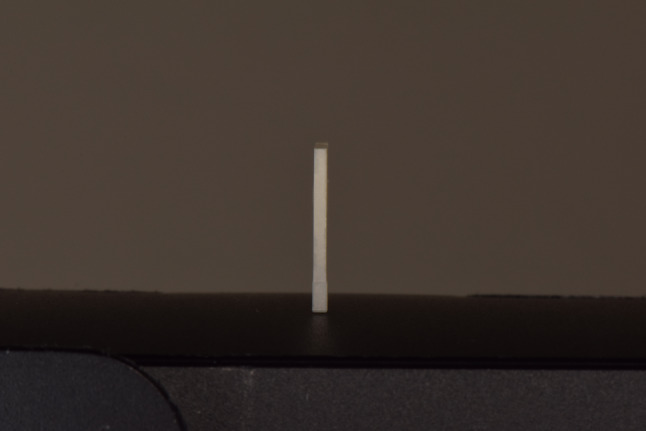
1 mm thickness Zirconia sample

After sintering, the specimen’s ultimate thickness was established by using silicon carbide abrasive papers (380,600-grit) under flowing water. Samples that did not meet the specified dimensions of 1 mm (0.05) (Fig. 1) thickness were discarded. Under flowing water, silicon carbide abrasive sheets of 1600 grit were used for 20 s apiece to provide the final polishing. Samples were then cleaned in an ultrasonic bath by using distilled water for 10 min and dried by using compressed air.

**Fig. 2:**
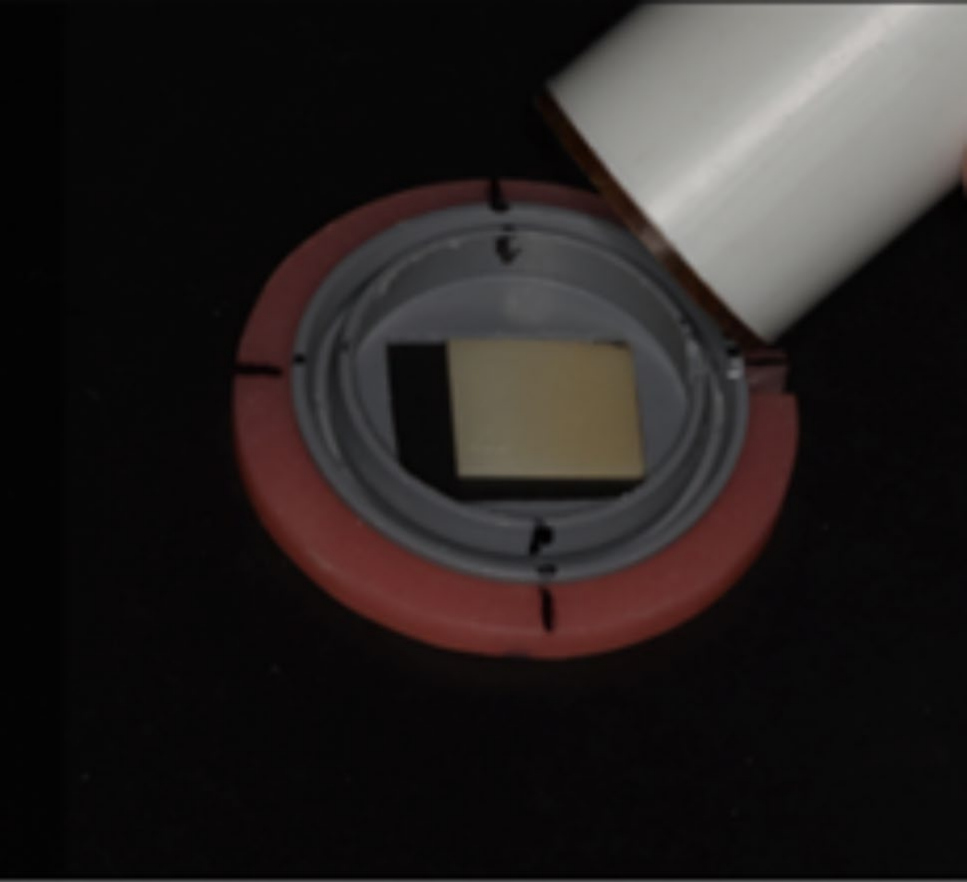
Zirconia specimen in a customized holder

Using a spectrophotometer (Agilent Cary 5000 UV-Vis-NIR spectrophotometer, Agilent Technologies, USA) calibrated with white and black calibration tiles (Fig. 2), the TP was measured using a customized holders. Spectrophotometer specifications were a light source of tungsten halogen visible and deuterium arc UV, a maximum scanning speed of UV-Vis 2,000 nm/min, a double beam of 8 Abs photometric range, and a wave length of 175–3300 nm. This was regarded as the T1 baseline. The L*a*b* values of each sample using average daylight illumination light source (D65) were measured according to the standards of the International Commission on Illumination (CIE). The TP was determined by comparing the sample’s color difference on a white and black background basis.

The L*, a*, and b* values were (L = 0.01, a = -0.02, and b = 0.01) for the black backgrounds and (L = 90.35, a = -1.31, and b = -0.27) for the white ones. After measuring each background three times with a 5-mm aperture, translucency parameter (TP_00_) values were determined by calculating the CIEDE2000 color difference formula (ΔTP_00_) between the color readings over the black and white backgrounds, according to the following equation [19]:

$${\text{T}\text{P}}_{00}=\sqrt{{\left(\frac{{L}_{B}^{{\prime }}-{L}_{W}^{{\prime }}}{{k}_{\text{L}}{S}_{\text{L}}}\right)}^{2}+{\left(\frac{{C}_{B}^{{\prime }}-{C}_{W}^{{\prime }}}{{k}_{\text{C}}{S}_{\text{C}}}\right)}^{2}+{\left(\frac{{H}_{B}^{{\prime }}-{H}_{W}^{{\prime }}}{{k}_{\text{H}}{S}_{\text{H}}}\right)}^{2}+{R}_{\text{T}}\left(\frac{{C}_{B}^{{\prime }}-{C}_{W}^{{\prime }}}{{k}_{\text{C}}{S}_{\text{C}}}\right)\left(\frac{{H}_{B}^{{\prime }}-{H}_{W}^{{\prime }}}{{k}_{\text{H}}{S}_{\text{H}}}\right)}$$

Where the subscripts “B” and “W” refer to color coordinates of each layer over the black and white backgrounds, respectively. The weighting functions, S_L_, S_C_, and S_H_ adjust the total color difference for variation in the location of the color difference pair in L′, a’, and b’ coordinates, and the parametric factors k_L_, k_C_, and k_H_ are correction terms for experimental conditions. In the present study, k_L_ = k_C_ = k_H_ = 1 was considered. Translucency differences (ΔTP_00_) were evaluated in accordance with the 50%:50% translucency perceptibility and acceptability (TPT_00_ = 0.62 and TAT_00_ = 2.62) thresholds [20, 21].

Following the T1/baseline measurement, all samples were thermally aged using a thermocycler (Thermocycler THE 1100 SD Mechatronik GmbH, Germany) throughout a range of 5 °C to 55 °C, with a dwell time of 30s and a transfer time of 10s, as per the guidelines of ISO 11,405. T2 = 10,000 thermal cycles (representing one year of clinical use), T3 = 30,000 thermal cycles (representing three years of clinical use), and T4 = 50,000 thermal cycles were performed (5 years of clinical use). At the end of each ageing cycle (T2, T3, and T4), the samples were removed from the distilled water, dried up with paper tissues. At each time point, TP was calculated by using the aforementioned formula.

SPSS version 25 was used for all statistical analyses (IBM-SPSS, Armonk, NY, USA). The descriptive statistics for the translucency profile at baseline and the translucency profile after thermocycling were found to be normally distributed, necessitating the use of parametric statistics. The Shapiro-Wilk (SW) test was utilised to inspect the normality of the estimated translucency values (SW = 0.996, *p* = .841). Comparing the L, a, and b values at baseline was done by using a one-way ANOVA. The Scheffe post-hoc compared differences among each of the four materials. The repeated measures ANOVA tested the differences between the materials at each of the different thermocycling intervals. Data analyses were evaluated at a significance level of *p* < .05 (CI 95%).

## Results

**Fig. 3:**
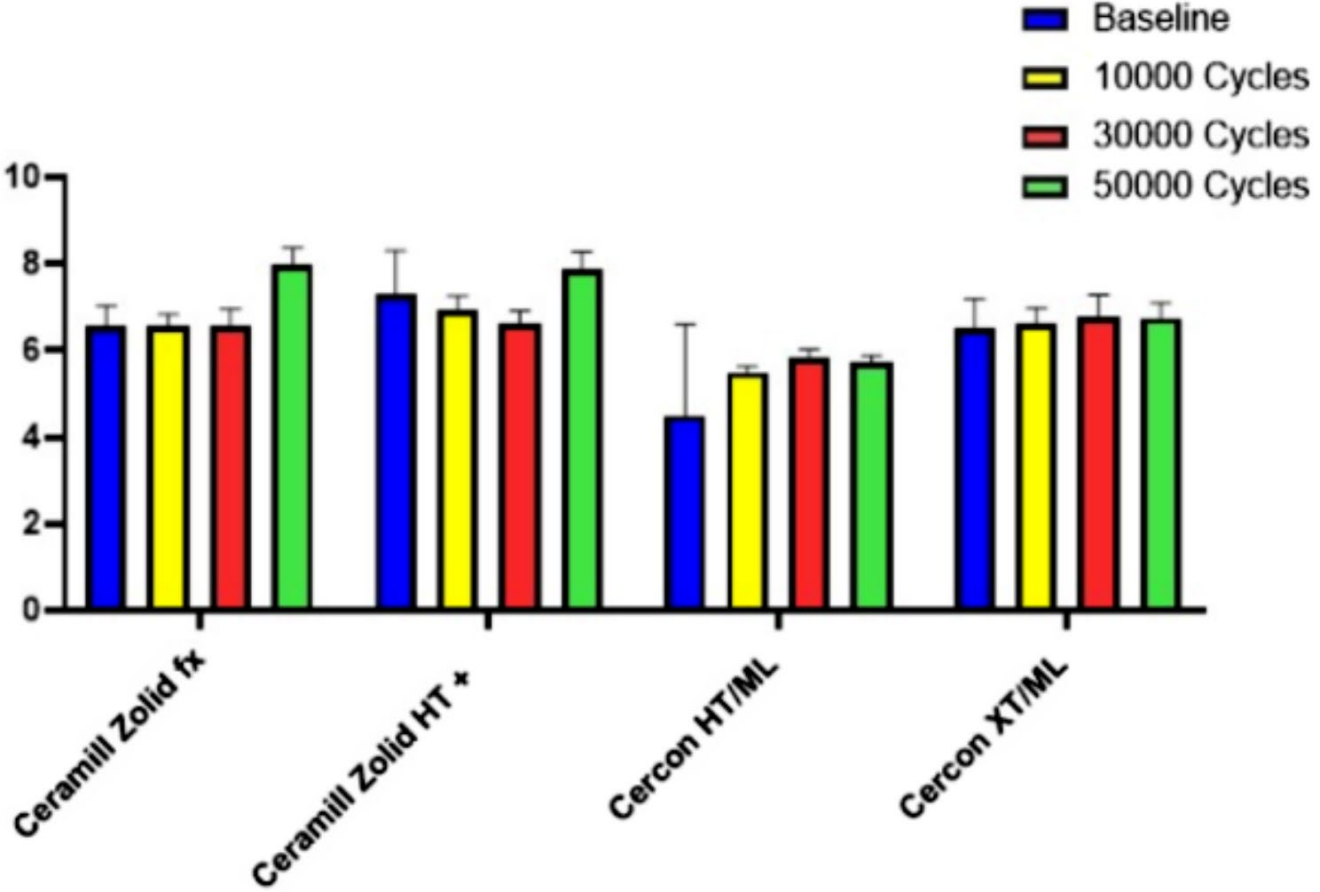
Impact of thermocycling within each material over the number of cycles

The two-way ANOVA showed significant differences between the materials at each of the different thermocycling intervals (*p* < .001) (Table 2). The Scheffe post-hoc test showed that there were significant differences among each of the four materials. At each of the time intervals, group 2 [CM-4Y] had the highest TP followed by group1 [CM-5Y], while, group 3 [CC-5Y] and group 4 [CC-4Y] had the least TP. There was statistically significant differences between the zirconia samples at each thermocycling interval (*p* = .003) (Table 2). Scheffe’s post hoc test showed that at baseline and 10,000 cycles, the TP of group 2 [CM-4Y] was substantially greater than that of the other materials. Group1 [CM-5Y] and group 3 [CC-5Y] did not differ significantly at baseline, 10,000, or 30,000 cycles. Group 4 [CC-4Y] showed a significantly lower TP than all other materials at baseline and each thermocycling interval. At 50,000 cycles, there was a statistically significant difference (*p* = .701) between the TP of the four materials (Fig. 3).

**Table 2:** Comparison of Translucency Profile of the different Materials at different intervals of thermocycling

		Mean	Std. Dev.	95% Confidence Interval		F*	Sig
				Lower Bound	Upper Bound		
Baseline	Ceramill Zolid fx ^a^	6.6109	0.40393	6.5119	6.6862	88.546	< 0.001**
	Ceramill Zolid HT + ^b^	7.0581	0.75631	6.9731	7.4028		
	Cercon XT/ML ^a^	6.3447	0.58895	6.1041	6.4931		
	Cercon HT/ML ^c^	4.5013	1.8949	4.3822	4.9598		
10,000 Cycles	Ceramill Zolid fx ^a^	6.5830	0.28230	6.5110	6.6003	478.414	< 0.001**
	Ceramill Zolid HT + ^b^	7.0106	0.24320	6.9243	7.0040		
	Cercon XT/ML ^a^	6.4702	0.34618	6.3621	6.6910		
	Cercon HT/ML ^c^	5.4160	0.14220	5.4003	5.5288		
30,000 Cycles	Ceramill Zolid fx ^a^	6.5682	0.36831	6.4685	6.7184	107.702	< 0.001**
	Ceramill Zolid HT + ^a^	6.4432	0.28424	6.4001	6.5720		
	Cercon XT/ML ^a^	6.5801	0.49050	6.4911	6.7006		
	Cercon HT/ML ^b^	5.8115	0.17881	5.8001	5.8713		
50,000 Cycles	Ceramill Zolid fx ^a^	8.0016	0.39545	7.9218	8.1394	861.432	< 0.001**
	Ceramill Zolid HT + ^b^	7.6348	0.35010	7.5581	7.7061		
	Cercon XT/ML ^d^	6.3852	0.41140	6.2637	6.6619		
	Cercon HT/ML ^c^	5.6052	0.17178	5.5900	5.6218		

*Calculated by using repeated measures ANOVA

** Differences are significant at *p* < .05

a, b,c, d: Differences in superscript indicate significant difference at *p* < .05

From baseline to 30,000 cycles, TP decreased significantly for both group1 [CM-5Y] (*p* = .004) and group 2 [CM-4Y] (*p* = .006). However, TP increased significantly at 50,000 cycles for both materials. There was a statistically significant rise in the translucency parameter from the starting point to 30,000 cycles in both group 3 [CC-5Y], and group 4 [CC-4Y]. However this began to diminish at 50,000 cycles (*P* < .001) (Table 2).

## Discussion

The hypothesis of this study stating that there will be no significant variations in the translucency of zirconia between the third and fourth generations at different time intervals was rejected. The results of the current study revealed that, at baseline, the third generation zirconia, surprisingly showed statistically significantly less TP compared to fourth generation. Although third and fourth generations of zirconia are classified as monolithic zirconia materials, they differ in their composition [15].

This result is partially compatible with Shen et al., 2019’s study, in which the authors reported the lowest TP values after 50,000 TC and coffee immersion. Hydrothermal aging may cause changes to the Y-TZP crystal structure and promote reactions within the grain boundaries, which could affect the material’s opacity by increasing the difference in refractive indexes between the different phases [22]. This finding contradicts previous studies [21, 23]. The authors anticipated that the yttrium content would be increased in the third generation, resulting in larger cubic-form zirconia grains with smaller scattering grain-boundary areas, which would improve zirconia’s translucency due to optical anisotropy.

The modest rise in zirconia’s TP after 50,000 cycles of ageing; could be due to grain size and the need for a transformation to monoclinic form at the surface. Kim and Kim discovered that autoclaving lithium disilicate and monolithic zirconia for up to 10 h significantly improved their translucency [21]. Sulaiman et al. reported a similar increase in translucency for partly stabilised zirconia after 96 h of acidic ageing in an incubator at 37 °C [24].

The findings of this investigation support the manufacturer’s assertion that the novel monolithic multilayer zirconia enhances translucency. On the other hand, they still exhibited a lower TP value than glass-based ceramics due to the much reduced light transmission through zirconia compared to glass-based ceramics. A study identified a TP value of 19 for lithium disilicate glass-ceramic [23], while Aljanobi G and Al-Sowygh ZH observed 16.9, which are nearly double the outcomes of the current study’s zirconia [25].

Translucency is an important requirement for mimicking the appearance of natural teeth, and it has been identified as a crucial aspect of material selection [9, 16]. The crystalline content, grain size, core colour, oral environment conditions, and microstructural differences determine the difference between the TP values of the materials [15]. With claims of greater translucency properties regarding newer monolithic zirconia ceramic generations, limited research has been available evaluating their translucency and the effects of long-term aging [16]. This research set out to evaluate the effect of aging on the translucency of third and fourth-generation zirconia at a thickness of 1 mm. Disc-shaped specimens with a thickness of 1 mm were created to ease optical measurements on a flat surface. Before implementing a novel material in dentistry, it is necessary to test the material in settings that mimic the oral environment [15–18].

The chroma variation could be the cause for the decrease in translucency in the third generation. The present results also come in agreement with another study [26]. This study’s results contradict the findings of a previous study, which concluded that all fourth-generation zirconia materials exhibited moderate translucency [22]. Cho et al., 2020, also concluded that the translucency of zirconia specimens tended to rise when yttrium concentration increased [27].

The studies for each generation of zirconia showed a diverse range of TP values, which contributed primarily to methodological differences owing to sample thickness, sintering process, different white and black background values used, and measuring tools [20, 24, 28].

The present results also agree with De Souza et al. 2017 [26] and Kelch et al., 2019 [28], who analysed the influence of hydrothermal ageing on zirconia and stated that it affects monoclinic phase content and surface topography. The present results coincide with those of Kurt and Bal 2019 who evaluated the effect of hydrothermal aging on the translucency of zirconia and found that for stabilized zirconia, it decreased significantly after aging. The pores formed after aging cause an increase in the scattering of incident light that decreases translucency. Furthermore, the presence of cubic zirconia accelerates tetragonal to monoclinic transformation [20].

It is essential to understand how structural surface changes subsequent to low thermal degradation (LTD) can influence the optical properties of zirconia dental restorations after being exposed to the oral environment for a long period of time, as reported by Angela et al., 2016 [29].

Consequently, it is challengingto compare the results of different investigations. However, most studies concur that the translucency of the third and fourth generations of zirconia is superior to that of the first and second generations.

This research highlights the influence of different zirconia generations on the translucency of aesthetic restorations after intraoral aging. At each time interval, group 2: [CM-4Y] had the highest translucency values followed by group1: [CM-5Y], group 3: [CC-5Y] and group 4: [CC-4Y] had the least translucency values. This enlightens clinicians about the fact that zirconia microstructure and yttrium content can influence translucency outcomes. Therefore, recommendations for each type of zirconia restoration in different clinical situations are essential. For high aesthetic needs; group 2: [CM-4Y] Ceramill Zolid HT + can be the material of choice, and group 4: [CC-4Y] Cercon HT/ML (4Y 4th generation zirconia) can be used in less aesthetic areas with dark dentin stumps.

The small sample size of this study is its primary limitation, which can be justified by the fact that the 5% margin of error assumption was used in its calculation. Lower-margin errors should be considered in future research. This study had some limitations that also need to be assessed for a better understanding of the translucency of monolithic zirconia clinical studies applications and the influence of cement on long-term colour stability. In addition, improved imaging and microstructure analysis.

## Conclusions

Within the limitations of this in-vitro study, it was concluded:

1. Different generations of zirconia display different translucency patterns.
2. Thermocycling has significant effect on the translucency of both third and fourth-generation zirconia.
3. At each of the time intervals, group 2: [CM-4Y] had the highest TP, followed by group1 [CM-5Y], while, group 3 [CC-5Y] and group 4 [CC-4Y] had the least TP.

## Data Availability

All data supporting the findings of this study are available within the paper.

## Abbreviations

5Y: 5 mol% yttria-stabilized zirconia polycrystalline ceramic
4Y: 4 mol% yttria-stabilized zirconia polycrystalline ceramic
3Y-TZP: 3-mol%-yttria-stabilized tetragonal zirconia polycrystals
PSZ: Partially stabilized
FSZ: Fully stabilized
MP: Mega Pascal
TP: Translucency parameter
CR: Contrast ratio
alpha (α): alpha
(β): beta
SPSS: Statistical Package for the Social Sciences
